# Gene-wide significant association analyses of *DNMT1* genetic variants with Parkinson’s disease

**DOI:** 10.3389/fgene.2023.1112388

**Published:** 2023-03-06

**Authors:** Jian-Yong Wang, Lei Cui, Hong-Yi Shi, Ling-Hao Chen, Ren-Wei Jin, Xiao-Xia Jiang, Zhu-Ling Chen, Jian-Hong Zhu, Xiong Zhang

**Affiliations:** ^1^ Department of Neurology, Institute of Geriatric Neurology, The Second Affiliated Hospital and Yuying Children’s Hospital, Wenzhou Medical University, Wenzhou, Zhejiang, China; ^2^ Department of Preventive Medicine, Institute of Nutrition and Diseases, Wenzhou Medical University, Wenzhou, Zhejiang, China

**Keywords:** Parkinson’s disease, DNMT1, polymorphism, association, methylation

## Abstract

**Background:** DNA methylation plays an important role in Parkinson’s disease (PD) pathogenesis. DNA methyltransferase 1 (DNMT1) is critical for maintaining DNA methylation in mammals. The link between *DNMT1* polymorphisms and PD remains elusive.

**Methods:** The *DNMT1* gene contained a total of 28 single nucleotide polymorphisms (SNPs). Four representing tag-SNPs (rs16999593, rs2162560, rs11880553, and rs9305012) were identified and genotyped in a Han Chinese population comprising 712 PD patients and 696 controls. Association analyses were performed at gene-wide significance (*p* < 1.8 × 10^−3^).

**Results:** Rs9305012, but not the other 3 tag-SNPs, was gene-wide significantly associated with PD risk (*p* = 0.8 × 10^−3^). The rs9305012/C was a protective allele against PD (*p* = 1.5 × 10^−3^, OR 0.786, 95% CI 0.677–0.912). No significant association was observed in individual genders or PD subtypes. Haplotypes of the 4 tag-SNPs showed a significant overall distribution difference between PD patients and controls (*p* < 1 × 10^−4^). The 3-allele ACC module in the order of rs2162560, rs11880553, and rs9305012 was the highest-risk haplotype associated with PD (*p* < 1 × 10^−4^, OR 2.439, 95% CI 1.563–3.704). Rs9305012 displayed certain probability to affect transcription factor binding and target gene expression based on functional annotation analyses.

**Conclusion:** The *DNMT1* variant rs9305012 together with its haplotypes may gene-wide significantly modulate PD susceptibility. Our results support a role of DNMT1 in PD pathogenesis and provide novel insights into the genetic connection in between.

## Introduction

Parkinson’s disease (PD) is the second most common neurodegenerative disorder after Alzheimer’s disease, with bradykinesia, rest tremor, and rigidity as its cardinal motor manifestations (Postuma et al., 2015). Although its unequivocal etiology remains unknown, PD is believed to be caused by genetic factors, environmental exposures, and their interactions (Kalia and Lang, 2015; Fan et al., 2021). Epigenetic modulation such as DNA methylation may be a link connecting genetic and environmental mechanisms (Zhang et al., 2021). *SNCA* encodes the core PD-associated protein, α-synuclein. The protein expression could be mediated by the CpG island methylation in *SNCA* intron 1, and the methylation level was reduced in the lesioned brain areas of PD patients (Jowaed et al., 2010; Matsumoto et al., 2010). Indeed, DNA methylation has been considered to play an important role in PD pathogenesis (Wullner et al., 2016).

DNA methylation patterns are established and maintained by DNA methyltransferases (DNMTs). DNMT3A and DNMT3B are responsible for the establishment of DNA methylation during embryonic development, while DNMT1 is critical for maintaining the methylation patterns established early in development (Li and Zhang, 2014). Amongst, DNMT1 is abundantly expressed in human brain, and its expression level was reported to be reduced with a global DNA hypomethylation in postmortem brain samples of PD patients (Desplats et al., 2011). DNMT1 expression is also noted to be downregulated in multiple PD cellular and animal models, which cause a hypomethylation in long interspersed nuclear element 1, a global DNA methylation indicator (Zhang et al., 2021). In addition, a system based on Clustered Regularly Interspaced Short Palindromic Repeats (CRISPR)-deactivated Cas9 fused with the catalytic domain of DNMT3A appears to be effective in modulating *SNCA* intron 1 methylation and downregulating *SNCA* expression (Kantor et al., 2018; Tagliafierro et al., 2019).

PD is heterogeneous in clinical presentation, treatment responsiveness, and underlying pathology (Mestre et al., 2021). PD subtypes, including postural instability gait disorder (PIGD), tremor dominant (TD), and intermediate, are significantly different in motor and non-motor symptoms (Jankovic et al., 1990; Wang et al., 2020). In this study, we aimed to understand whether and how DNMT1 genetic variant, individually or in form of haplotype, are significantly associated with risk for PD and PD subtypes by analyzing tag-single nucleotide polymorphisms (tag-SNPs) in a large Chinese cohort.

## Materials and methods

### Subjects

A total of 1,408 subjects of Han Chinese ethnicity were enrolled in this study, including 712 sporadic PD patients and 696 controls. PD patients were diagnosed by two movement disorder neurologists according to the United Kingdom Parkinson’s Disease Society Brain Bank Criteria (Hughes et al., 1992). Patients with secondary and atypical parkinsonism or with a family history of PD were excluded. Controls were healthy volunteers based on routine physical examinations. Those with history of neurological and psychiatric disorders were excluded. PD subtypes of TD, PIGD, and intermediate were classified based on the Unified PD rating scale (UPDRS) items as previously described (Jankovic et al., 1990; Wang et al., 2020). Patients were not discriminated for treated or newly diagnosed; the UPDRS evaluation was performed during “OFF” state (Bordelon et al., 2011). All patients and controls participating in the study signed written informed consents. The study was approved by the Ethics Committee of the Second Affiliated Hospital and Yuying Children’s Hospital, Wenzhou Medical University.

### Tag-SNPs

A total of 28 variants in *DNMT1* were extracted using the HapMap project with minor allele frequency (MAF) ≥ 0.1 in Han Chinese population from Beijing, China. Four linkage disequilibrium blocks, covering the 28 *DNMT1* variants, were identified according to Haploview v.4.2 (Barrett et al., 2005) with pairwise tagging and *r* ^2^ ≥ 0.8 (Supplementary Figure S1). In detail, block 1 included rs16999593; block 2 included rs2162560, rs2288349, rs6511677, rs7253062, and rs8101626; block 3 included rs11880553, rs8112801, rs759920, rs2290684, rs8101866, rs6511685, rs2114724, rs2228611, and rs11880388; block 4 included rs9305012, rs2241531, rs4804490, rs4804494, rs10407514, rs10854076, rs10418707, rs10423341, rs8111085, rs8112895, rs11672909, rs10420321, and rs2288350. Four representing SNPs, with one in each block and preferentially with restriction endonuclease site available, were selected as tag-SNPs for further genotyping and analyses. These were rs16999593 (T/C), rs2162560 (G/A), rs11880553 (C/T), and rs9305012 (T/C).

### Genotyping

Genomic DNA was extracted from human peripheral blood samples. The four tag-SNPs were identified using polymerase chain reaction-restriction fragment length polymorphism as previously described (Zhang et al., 2014). Primer pairs, restriction enzymes (all from New England BioLabs, Beverly, MA, United States), and fragment lengths of the SNPs were listed in Supplementary Table S1. Polymerase chain reactions (PCRs) were carried in a total volume of 25 μL, which contained 0.1 μg of DNA, 0.5 μM of each primer, and 12.5 μL of 2× PCR Mastermix (Tiangen, Beijing, China) according to the manufacturer’s protocol. The annealing temperature was 49°C for rs16999593, 57°C for rs2162560, 56°C for rs11880553, and 52°C for rs9305012. Thirty samples were verified by direct sequencing (BGI Tech, Shanghai, China) for each SNP. All the results were in consistency with those of enzymatic genotyping.

### Data analysis

Analyses were performed using statistical package of Predictive Analytics Software (PASW, version 19.0). Hardy-Weinberg equilibrium calculated by Chi square test was used to evaluate the genotype distribution. Kolmogorov-Smirnov test was used to evaluate the normality. Mann-Whitney *U* test was used to assess the difference in age between the cases and controls. Chi square test was used to assess the differences in gender, genotype, and allele frequencies between the two groups. Multivariate analysis was performed by binary logistic regression model with gender, age, and genotypes as covariates. The haplotype construction and analysis were performed using the SNPStats Online Version (https://www.snpstats.net/start.htm). The highest-risk haplotype was identified by a backward elimination method as stated previously (Francis et al., 2007). Bonferroni correction rather than the simple correction was used for multiple testing for SNPs in linkage disequilibrium to best prevent false-positive findings (Nyholt, 2004). Significance for association between PD and variants was considered when reaching a gene-wide *p* < 1.8 × 10^−3^. For the remaining comparisons, a two-tailed *p* < 0.05 was considered significant.

### Function annotation

The variants were annotated for functions as previously described (Fan et al., 2022). In brief, potential causal links to diseases were annotated using HaploReg (http://pubs.broadinstitute.org/mammals/haploreg/haploreg.php). Genetic variations in regulatory elements in intergenic regions of the human genome were annotated using RegulomeDB (http://www.regulomedb.org/). The expression quantitative trait locus (eQTL), splicing quantitative trait locus (sQTL), and expression data were analyzed using the GTEx Portal (https://gtexportal.org/).

## Results

### Demographic and clinical data

PD patients included 373 males and 339 females; controls included 374 males and 322 females. The median age of PD patients and controls was 66 (interquartile range, 59–73) and 60 (interquartile range, 52–72) years old, respectively. The PD and control groups were comparable in gender (*p* > 0.05), but not in age (*p* < 0.05). The analyses were thus performed with age and sex adjustments. Records available showed that a total of 535 PD patients were classified with PD subtypes, including 290 TD, 179 PIGD, and 66 intermediates. The intermediate subtype was not included for individual subtype analysis due to insufficient number. No difference was shown in UPDRS scores or Hoehn & Yahr stage between the PIGD and TD subtypes (Table 1).

**TABLE 1:** Demographic and clinical characteristics.

	PD	Control	*P*	TD	PIGD	Intermediate	*P* ^c^
Subject n (%)	712 (50.6)	696 (49.4)	—	290 (54.2)	179 (33.5)	66 (12.3)	—
Age, median (IR)	66 (59–72)	60 (52–72)	< 0.001^a^	67 (60–73)	65 (58–73)	64 (56–71)	0.048^a^
Gender, F/M	339/373	322/374	0.612^b^	135/155	81/98	31/25	0.784^b^
UPDRS-Total	—	—	—	38 (26–56)	35 (26–53)	45 (31–74)	0.523^a^
UPDRS-III	—	—	—	24 (15–36)	22 (15–32)	29 (18–46)	0.109^a^
Hoehn & Yahr stage				2 (1–2.5)	2 (1–2.5)	2 (1.5–3)	0.230^a^

^a^ Analyzed by Mann-Whitney *U* test.

^b^ Analyzed by Chi square test.

^c^ Compared between the PIGD and TD subtypes. The intermediate subtype was not included for analyses due to insufficient number.

IR, interquartile range; PD, Parkinson’s disease; PIGD, postural instability and gait disturbance; TD, tremor-dominant; UPDRS, unified Parkinson’s disease rating scale.

### Association analysis of the *DNMT1* tag-SNPs with PD susceptibility

Frequencies of rs16999593, rs2162560, rs11880553, and rs9305012 in the controls met with Hardy-Weinberg equilibrium (*p* > 0.05). The variant rs9305012 displayed a gene-wide significant difference in genotype distribution between PD patients and controls (*p* = 0.8 × 10^−3^; Table 2). The rs9305012/C was a protective allele against PD (*p* = 1.5 × 10^−3^, OR 0.786, 95% CI 0.677–0.912). The other 3 tag-SNPs (rs16999593, rs2162560 and rs11880553) showed no gene-wide significant difference in both genotype distribution and allele frequency. We further analyzed rs9305012 by three genetic models (additive, dominant, and recessive), and found that the variant was associated with PD in both dominant model (*p* = 2.3 × 10^−4^, OR 0.616, 95% CI 0.476–0.797) and additive model (*p* = 6.3 × 10^−4^, OR 0.754, 95% CI 0.641–0.887; Supplementary Table S2).

**TABLE 2:** Genotype and allele frequencies of *DNMT1* tag-SNPs in PD patients and controls.

Tag-SNPs	Genotype, n (%)			*P* ^a^	Allele, n (%)		*P* ^a^	OR (95% CI)
rs16999593	TT	TC	CC		T	C		
Controls	376 (54.0)	284 (40.8)	36 (5.2)	6.7 × 10^−3^	1,036 (74.4)	356 (25.6)	2.2 × 10^−3^	0.758 (0.6345–0.905)
PD	442 (62.1)	247 (34.7)	23 (3.2)		1,131 (79.4)	293 (20.6)		
rs2162560	GG	GA	AA		G	A		
Controls	326 (46.8)	321 (46.1)	49 (7.0)	5.0 × 10^−3^	973 (69.1)	419 (30.1)	4.3 × 10^−3^	1.261 (1.076–1.479)
PD	296 (41.6)	334 (46.9)	82 (11.5)		926 (65.0)	498 (35.0)		
rs11880553	CC	CT	TT		C	T		
Controls	342 (49.1)	297 (42.7)	57 (8.2)	0.755	981 (70.5)	411 (29.5)	0.772	0.976 (0.829–1.149)
PD	362 (50.8)	289 (40.6)	61 (8.6)		1,013 (71.1)	411 (28.9)		
rs9305012	TT	TC	CC		T	C		
Controls	126 (18.1)	412 (59.2)	158 (22.7)	0.8 × 10^−3^*	664 (47.7)	728 (52.3)	1.5 × 10^−3^*	0.786 (0.677–0.912)
PD	184 (25.8)	391 (54.9)	137 (19.2)		759 (53.3)	665 (46.7)		

^a^ Adjusted with age and sex.

* *p* < 1.8 × 10^−3^.

CI, confidence interval; DNMT1, DNA methyltransferase 1; OR, odds ratio; PD, Parkinson’s disease; SNP, single nucleotide polymorphism.

### Haplotype analysis of the *DNMT1* tag-SNPs

Haplotypes were constructed following the order of rs16999593, rs2162560, rs11880553, and rs9305012. Haplotypes whose frequency less than 0.03 were excluded from analysis. A gene-wide significant difference was found in overall haplotype distribution between PD and control groups (*p* < 1 × 10^−4^). However, none of the 4-allele haplotypes displayed significant association with PD predisposition (Table 3). Based on the backward elimination model, a highest-risk haplotype towards PD was retrieved, that is, ACC in the order of rs2162560, rs11880553, and rs9305012 from the best 3-allele model (*p* < 1 × 10^−4^, OR 2.439, 95% CI 1.563–3.704; Table 4).

**TABLE 3:** Haplotype analysis of *DNMT1* tag-SNPs in PD patients and controls.

Haplotype^a^	PD, n (%)	Control, n (%)	*P* ^b^	OR (95% CI)
TACT	308.3 (21.6)	309.2 (22.2)	—	1.000
TGCC	285.4 (20.0)	307.1 (22.1)	0.190	0.847 (0.654–1.087)
TGTT	287.1 (20.2)	269.9 (19.4)	0.860	1.020 (0.787–1.333)
CGCC	202.2 (14.2)	256.3 (18.4)	0.018	0.719 (0.546–0.943)
TACC	77.7 (5.5)	34.6 (2.5)	0.017	1.923 (1.124–3.333)
TGCT	68.8 (4.8)	38.2 (2.7)	0.062	1.563 (0.980–2.500)
TGTC	42.3 (3.0)	35.9 (3.3)	0.610	0.870 (0.513–1.471)
Total	1,424 (100)	1,392 (100)	< 1 × 10^−4^*	—

^a^ Haplotype alleles were in the order of rs16999593, rs2162560, rs11880553, and rs9305012. Haplotypes with frequency <3% in both patients and controls were excluded from the analysis.

^b^ Adjusted with age and sex.

* *p* < 1.8 × 10^−3^.

CI, confidence interval; DNMT1, DNA methyltransferase 1; OR, odds ratio; PD, Parkinson’s disease; SNP, single nucleotide polymorphism.

**TABLE 4:** The highest-risk haplotype analysis of *DNMT1* in association with PD.

n	Haplotype^a^	PD, n (%)	Control, n (%)	*P* ^b^	OR (95% CI)
4	TACC	77.7 (5.5)	34.6 (2.5)	0.017	1.923 (1.124–3.333)
3	-ACC	110.9 (7.8)	50.9 (3.7)	< 1 × 10^−4^*	2.439 (1.563–3.704)
2	-A-C	128.2 (9.0)	82.5 (5.9)	1.6 × 10^−3^*	1.852 (1.266–2.703)

^a^ Haplotype alleles were in the order of rs16999593, rs2162560, rs11880553, and rs9305012. Haplotypes with frequency <3% in both patients and controls were excluded from the analysis. A hyphen indicates the eliminated variant at that position.

^b^ Adjusted with age and sex.

* *p* < 1.8 × 10^−3^.

CI, confidence interval; DNMT1, DNA methyltransferase 1; OR, odds ratio; PD, Parkinson’s disease.

### Gender- and subtype-stratified analyses of the *DNMT1* variants

PD association was analyzed separately in male and female. The variants rs16999593, rs2162560, and rs11880553 remained not to be associated with PD in either male or female. The rs9305012 showed no more gene-wide significant association with PD in individual genders (Supplementary Table S3).

We then analyzed whether the tag-SNPs or their haplotypes were associated with the TD or PIGD subtype. Results showed that none of the SNPs was different in genotype distribution or allele frequency between controls and the PD subtypes (Table 5). Analyses of the 4-allele haplotypes also suggested no significant difference between controls and either of the PD subtypes (Table 6).

**TABLE 5:** Genotype and allele frequencies of *DNMT1* tag-SNPs in PD subtypes.

Tag-SNPs	Genotype, n (%)			*P* ^a^	Allele, n (%)		*P* ^a^	OR (95% CI)
rs16999593	TT	TC	CC		T	C		
Controls	376 (54.0)	284 (40.8)	36 (5.2)		1,036 (74.4)	356 (25.6)		
TD	183 (63.1)	96 (33.1)	11 (3.8)	0.059	462 (79.7)	118 (20.3)	0.028	0.765 (0.602–0.971)
PIGD	99 (55.3)	75 (41.9)	5 (2.8)	0.444	272 (76.0)	86 (24.0)	0.557	0.922 (0.703–1.209)
rs2162560	GG	GA	AA		G	A		
Controls	326 (46.8)	321 (46.1)	49 (7.0)		973 (69.1)	419 (30.1)		
TD	117 (40.3)	142 (49.0)	31 (10.7)	0.067	376 (64.8)	204 (35.2)	0.032	1.257 (1.020–1.549)
PIGD	71 (39.7)	87 (48.6)	21 (11.7)	0.050	229 (64.0)	129 (36.0)	0.029	1.315 (1.029–1.680)
rs11880553	CC	CT	TT		C	T		
Controls	342 (49.1)	297 (42.7	57 (8.2)		981 (70.5)	411 (29.5)		
TD	152 (52.4)	114 (39.3)	24 (8.3)	0.644	418 (72.1)	162 (27.9)	0.577	0.940 (0.756–1.168)
PIGD	90 (50.3)	75 (41.9)	14 (7.8)	0.976	255 (71.2)	103 (28.8)	0.830	0.973 (0.752–1.258)
rs9305012	TT	TC	CC		T	C		
Controls	126 (18.1)	412 (59.2)	158 (22.7)		664 (47.7)	728 (52.3)		
TD	67 (23.1)	161 (55.5)	62 (21.4)	0.164	295 (50.9)	285 (49.1)	0.191	0.877 (0.720–1.067)
PIGD	43 (24.0)	109 (60.9)	27 (15.1)	0.032	195 (54.5)	163 (45.5)	0.018	0.755 (0.597–0.953)

^a^ Adjusted with age and sex; compared with controls respectively; *p* < 1.8 × 10^−3^ was considered gene-wide significant.

CI, confidence interval; DNMT1, DNA methyltransferase 1; OR, odds ratio; PD, Parkinson’s disease; PIGD, postural instability and gait disturbance; SNP, single nucleotide polymorphism; TD, tremor-dominant.

**TABLE 6:** Haplotype analysis of *DNMT1* tag-SNPs in PD subtypes and controls.

PIGD					TD				
Haplotype^a^	PIGD, n (%)	Control, n (%)	*P* ^b^	OR (95% CI)	Haplotype^a^	TD, n (%)	Control, n (%)	*P* ^b^	OR (95% CI)
TACT	81.5 (22.8)	309.2 (22.2)	—	1.00	TGCC	121.3 (20.9)	307.1 (22.1)	—	1.00
TGCC	68.1 (19.0)	307.1 (22.1)	0.12	0.73 (0.49–1.09)	TACT	122.2 (21.1)	309.2 (22.2)	0.51	1.12 (0.80–1.57)
TGTT	76.6 (21.4)	269.9 (19.4)	0.77	0.94 (0.63–1.40)	TGTT	109.2 (18.8)	269.9 (19.4)	0.61	1.09 (0.78–1.53)
CGCC	58.1 (14.2)	256.3 (18.4)	0.12	0.71 (0.46–1.09)	CGCC	84.6 (14.6)	256.3 (18.4)	0.44	0.87 (0.61–1.24)
TGTC	4.3 (1.2)	35.9 (3.3)	0.14	0.38 (0.11–1.38)	TACC	36.7 (6.3)	34.6 (2.5)	0.002	2.67 (1.46–4.90)
Total	358 (100)	1,392 (100)	0.09	—	TGTC	22.6 (3.9)	35.9 (3.3)	0.63	1.18 (0.60–2.31)
					TGCT	25.9 (4.5)	38.2 (2.7)	0.05	1.82 (1.00–3.32)
					Total	580 (100)	1,392 (100)	0.015	—

^a^ Haplotype alleles were in the order of rs16999593, rs2162560, rs11880553, and rs9305012. Haplotypes with frequency <3% in both patients and controls were excluded from the analysis.

^b^ Adjusted with age and sex; compared with controls; *p* < 1.8 × 10^−3^ was considered gene-wide significant.

CI, confidence interval; DNMT1, DNA methyltransferase 1; OR, odds ratio; PD, Parkinson’s disease; PIGD, postural instability and gait disturbance; SNP, single nucleotide polymorphism; TD, tremor-dominant.

### Function annotation of rs9305012

**FIGURE 1:**
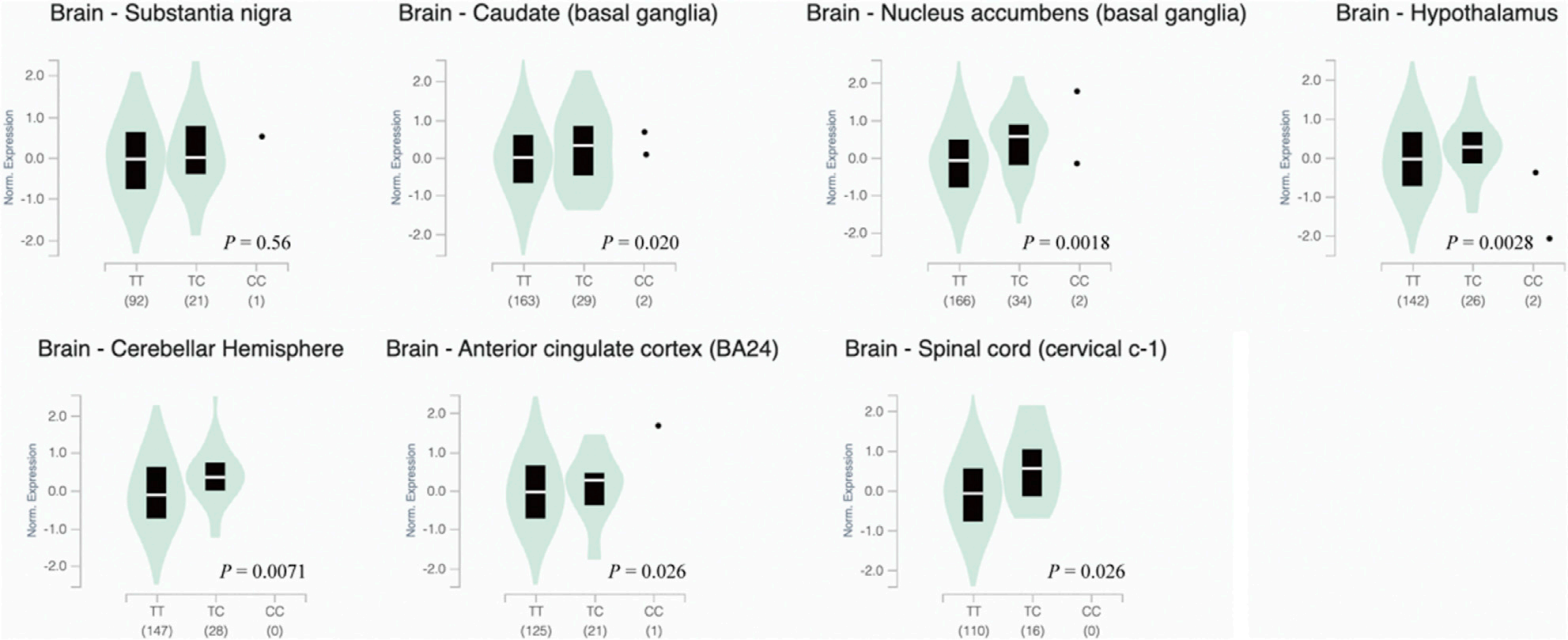
Violin plots of *P2RY11* expression in human brain tissues carrying different *DNMT1* rs9305012 genotype. Data were obtained from GTEx. Significance was considered at *p* < 0.05. The *p* value was from a *t*-test that compares observed normalized effect size to a null one of 0. Normalized effect size was the slope of the linear regression of normalized expression data versus the three genotype categories using single-tissue eQTL analysis. DNMT1, DNA methyltransferase 1; eQTL, expression quantitative trait locus; P2RY11, purinergic receptor P2Y, G-protein coupled, 11.

HaploReg, RegulomeDB, and GTEx were used for function annotation of the PD-associated variant. Based on the HaploRreg, rs9305012 was predicated to change the motifs of SP1 and Sp4. In RegulomeDB, rs9305012 was predicted with a score of 4. The score represents how the variant may affect transcription factor binding and/or DNase peak, and scores higher than 3 indicate a relatively low possibility with regulatory function (Luciano et al., 2018). By using GTEx, rs9305012 was suggested to be an eQTL of *P2RY11* (full name: purinergic receptor P2Y, G-protein coupled, 11). Affected brain tissues included spinal cord, cerebellar hemisphere, hypothalamus, anterior cingulate cortex, nucleus accumbens, and caudate (*p* < 0.05; Supplementary Figure S2). Further analysis using transcriptomic data from GTEx showed that individuals carrying the rs9305012/TT genotype displayed significantly (*p* < 0.05) lower *P2RY11* expression in caudate, nucleus accumbens, hypothalamus, cerebellar hemisphere, anterior cingulate cortex, and spinal cord, but not in substantia nigra, compared to the TC and CC genotypes (Figure 1). The sQTL analysis showed that rs9305012 was associated with the intron-excision ratio of *EIF3G* (full name: eukaryotic translation initiation factor 3 subunit G; only thyroid data were available; Supplementary Figure S3). In addition, the SNPs in linkage disequilibrium with rs9305012 are also located in the *DNMT1* introns except for rs8111085, which was a benign missense variant based on NCBI dbSNP.

## Discussion

DNMT1 plays an important role in methylation regulation and PD pathogenesis and may connect environmental impacts to the disease (Desplats et al., 2011; Li and Zhang, 2014; Zhang et al., 2021). Its genetic variations are thus likely involved in PD liability. By investigating four *DNMT1* tag-SNPs in a large Chinese population, we demonstrate that the variant rs9305012, overall 4-allele haplotype distribution, and the 3-allele ACC module are gene-wide significantly associated with PD risk. The significant association is not observed in the variants rs16999593, rs2162560, and rs11880553.

*DNMT1* has been genetically reported to associate with a number of diseases, including coronary artery disease (Peng et al., 2014), and gastric and breast cancers (Li et al., 2017). In contrast, not much is known regarding its genetic association with PD susceptibility, although the enzyme *per se* has been suggested to participate in PD pathogenesis (Desplats et al., 2011; Zhang et al., 2021). There is only one study showing that *DNMT1* variants, rs2162560 and rs759920 (in linkage disequilibrium with rs11880553), are not associated with PD risk in a Brazilian cohort containing 522 subjects (Pezzi et al., 2017). These results are in line with the current findings. Interestingly, there are multiple such kind studies for *DNMT3B*, although the results appear to be mixed (Chen et al., 2017; Pezzi et al., 2017; Pan et al., 2018). The significant association of rs9305012 with PD risk is suggested in both additive and dominant models, suggesting a dominant role of the disease-susceptible T allele. These findings together with the haplotype results are acquired after the gene-wide Bonferroni correction and should be valid in general since such correction contributes to overcorrection of the inflated false-positive rate (Nyholt, 2004). In addition, rs9305012 appears to marginally associate with PD in a recent study of imputation and reanalysis using genome-wide association data (Rodrigo and Nyholt, 2021). Local environmental exposures may also contribute to the susceptibility since epigenetic regulations are prone to such factors (Ammal Kaidery et al., 2013).

Minor allele frequencies of the four variants in our cohort match with those of East Asians in 1,000 Genomes (23.0% vs. 18.4% for rs16999593/C; 32.6% vs. 25.2% for rs2162560/A; 29.2% vs. 35.2% for rs11880553/T; and 49.5% vs. 39.5% for rs9305012/C). All the variants are not associated with PD risk gender-specifically and are not independently associated with the PD subtypes. Thus, DNMT1 may not be related to hormone regulation and clinical manifestations in PD. Haplotype represents global gene structure and is more representative than single SNP (Barrett et al., 2005). The ACC haplotype in the order of rs2162560, rs11880553, and rs9305012 may be the core effect alleles towards higher susceptibility to PD.

The rs16999593 is a missense variant (p.His97Arg), but its clinical significance was evaluated as benign (Richards et al., 2015), which may be why this SNP is not associated with PD. The PD-associated rs9305012 is located in the *DNMT1* intron and can regulate gene expression of *P2RY11* and intron slicing of *EIF3G* as suggested by quantitative trait locus analyses. P2RY11 is a member of purinergic receptors and plays an important role in regulation of proliferation, apoptosis, and chemotaxis of lymphocytes, monocytes and polymorphonuclear granulocytes (Kornum et al., 2011). Interestingly, the regulation of rs9305012 on *P2RY11* expression appears to be significant in caudate, nucleus accumbens, hypothalamus, cerebellar hemisphere, anterior cingulate cortex, and spinal cord, but not in substantia nigra. The substantia nigra is the core pathological region of PD where dopaminergic neurons degenerate, while other regions such as the caudate nucleus and nucleus accumbens are involved in movement and reward controls (Prakash, 2022). These results indicate that such a regulation may not directly participate in the degeneration of dopaminergic neurons, but more likely involve in movement disorder and symptom development in PD. The rs9305012 is predicted to be binding motifs of transcription factors SP1 and SP4. This SNP displays a CpG site at presence of the C allele and theoretically may be methylated and affects the binding of transcription factors although the probability to be functional may be low based on its RegulomeDB score (Luciano et al., 2018). Overall, rs9305012 could likely be involved in PD pathogenesis by regulating target gene expression, but further experimental investigations are warranted.

The study contains several limitations. The lack of environmental exposure information limits us from analyzing such contribution to the *DNMT1* association with PD in this population; the lack of clinical data such as non-motor symptoms prevent us from performing the related analysis; the gene-wide Bonferroni correction may overcorrect the false-positive rate. The study also lacks experimental validation for the association, and potential participation analysis of other epigenetic factors.

In conclusion, the current study demonstrates in a Chinese population that the *DNMT1* variant rs9305012 and the ACC haplotype are gene-wide significantly associated with PD susceptibility. The rs9305012/C allele contributes a protection against PD. Our findings support a role of DNMT1 in PD pathogenesis and provide novel insights into the genetic connection in between.

## Data Availability

The original contributions presented in the study are included in the article/Supplementary Material, further inquiries can be directed to the corresponding authors.
