# Supplementary material for: Gene-wide significant association analyses of DNMT1 genetic variants with Parkinson’s disease
Source: Front Genet. 2023 Mar 6;14:1112388. doi: 10.3389/fgene.2023.1112388 (PMC10025298; doi:10.3389/fgene.2023.1112388)
Supplement: Supplementary file 1 [file Table1.DOCX]

**Table S1. PCR primers and RFLP products**

| Tag-SNPs | Enzyme | Primers (5’ to 3’) | PCR product, bp | RFLP size, bp |
| --- | --- | --- | --- | --- |
| rs16999593 | *NlaIII* | F: TGGCACATACCTCTAATC | 385 | TT: 330+55 |
|  |  | R: CCTGGCTAAAGTCAAAT |  | TC:385+330+55 |
|  |  |  |  | CC: 385 |
| rs2162560 | *HaeIII* | F: GCACAGTAGCTCATGCCTG | 424 | GG: 279+145 |
|  |  | R: GGAGGTTACAGTGAGCCAA |  | GA: 424+279+145 |
|  |  |  |  | AA: 424 |
| rs11880553 | *MspI* | F: GCTTGGCTGCTTGCTCTG | 226 | CC: 175+51 |
|  |  | R: AACTTGCGGTGATTAGGC |  | CT: 226+175+51 |
|  |  |  |  | TT: 226 |
| rs9305012 | *NlaIII* | F: TGAAGAGTTGCTGCTAAA | 180 | TT: 110+70 |
|  |  | R: TGAGGTATTGTGGGCTGT |  | TC: 180+110+70 |
|  |  |  |  | CC: 180 |

F, forward; PCR, polymerase chain reaction; R, reverse; RFLP, restriction fragment length polymorphism; SNP, single nucleotide polymorphism

**Table S2. Genetic model analysis of rs9305012**

| Model | Genotype | *P* ^a^ | OR (95% CI) |
| --- | --- | --- | --- |
| Recessive | TT + TC *vs.* CC | 0.086 | 0.794 (0.613-1.031) |
| Dominant | TT *vs.* TC + CC | 2.3 × 10^-4^ * | 0.616 (0.476-0.797) |
| Additive | TT *vs.* TC *vs.* CC | 6.3 × 10^-4^ * | 0.754 (0.641-0.887) |

^a^ Adjusted with age and sex.

* *P* < 1.8 × 10^-3^.

CI, confidence interval; OR, odds ratio

**Table S3. Genotype and allele frequencies of *DNMT1* tag-SNPs in male and female**

| Tag-SNPs | Genotype, n (%) | | | *P* ^a^ | Allele, n (%) | | *P* ^a^ | OR (95% CI) |
| --- | --- | --- | --- | --- | --- | --- | --- | --- |
| rs16999593 | TT | TC | CC |  | T | C |  |  |
| Controls (M) | 203 (54.3) | 157 (42.0) | 14 (3.7) |  | 563 (75.3) | 185 (24.7) |  |  |
| PD (M) | 227 (60.9) | 130 (34.9) | 16 (4.3) | 0.135 | 584 (78.3) | 162 (21.7) | 0.180 | 0.848 (0.666-1.080) |
| Controls (F) | 173 (53.7) | 127 (39.4) | 22 (6.8) |  | 473 (73.4) | 171 (26.6) |  |  |
| PD (F) | 215 (63.4) | 117 (34.5) | 7 (2.1) | 4.8 × 10^-3^ | 547 (80.7) | 131 (19.3) | 2.3 × 10^-3^ | 0.665 (0.513-0.864) |
| rs2162560 | GG | GA | AA |  | G | A |  |  |
| Controls (M) | 169 (45.2) | 182 (48.7) | 23 (6.1) |  | 520 (69.5) | 228 (30.5) |  |  |
| PD (M) | 150 (40.2) | 176 (47.2) | 47 (12.6) | 8.2 × 10^-3^ | 476 (63.8) | 270 (36.2) | 0.020 | 1.293 (1.041-1.605) |
| Controls (F) | 157 (48.8) | 139 (43.2) | 26 (8.1) |  | 453 (70.3) | 191 (29.7) |  |  |
| PD (F) | 146 (43.1) | 158 (46.6) | 35 (10.3) | 0.199 | 450 (66.4) | 228 (33.6) | 0.079 | 1.235 (0.976-1.562) |
| rs11880553 | CC | CT | TT |  | C | T |  |  |
| Controls (M) | 194 (51.9) | 144 (38.5) | 36 (9.6) |  | 532 (71.1) | 216 (28.9) |  |  |
| PD (M) | 196 (52.5) | 145 (38.9) | 32 (8.6) | 0.885 | 537 (72.0) | 209 (28.0) | 0.728 | 0.960 (0.767-1.203) |
| Controls (F) | 148 (46.0) | 153 (47.5) | 21 (6.5) |  | 449 (69.7) | 195 (30.3) |  |  |
| PD (F) | 166 (49.0) | 144 (42.5) | 29 (8.6) | 0.364 | 476 (70.2) | 202 (29.8) | 0.987 | 0.998 (0.787-1.266) |
| rs9305012 | TT | TC | CC |  | T | C |  |  |
| Controls (M) | 63 (16.8) | 233 (62.3) | 78 (20.9) |  | 359 (48.0) | 389 (52.0) |  |  |
| PD (M) | 94 (25.2) | 206 (55.2) | 73 (19.6) | 0.014 | 394 (52.8) | 352 (47.2) | 0.056 | 0.820 (0.668-1.005) |
| Controls (F) | 63 (19.6) | 179 (55.6) | 80 (24.8) |  | 305 (47.4) | 339 (52.6) |  |  |
| PD (F) | 90 (26.5) | 185 (54.6) | 64 (18.9) | 0.019 | 365 (53.8) | 313 (46.2) | 7.6 × 10^-3^ | 0.742 (0.596-0.923) |

^a^ Adjusted with age. *P* < 1.8 × 10^-3^ was considered gene-wide significant.

CI, confidence interval; DNMT1, DNA methyltransferase 1; F, female; M, male; OR, odds ratio; PD, Parkinson’s disease; SNP, single nucleotide polymorphism.


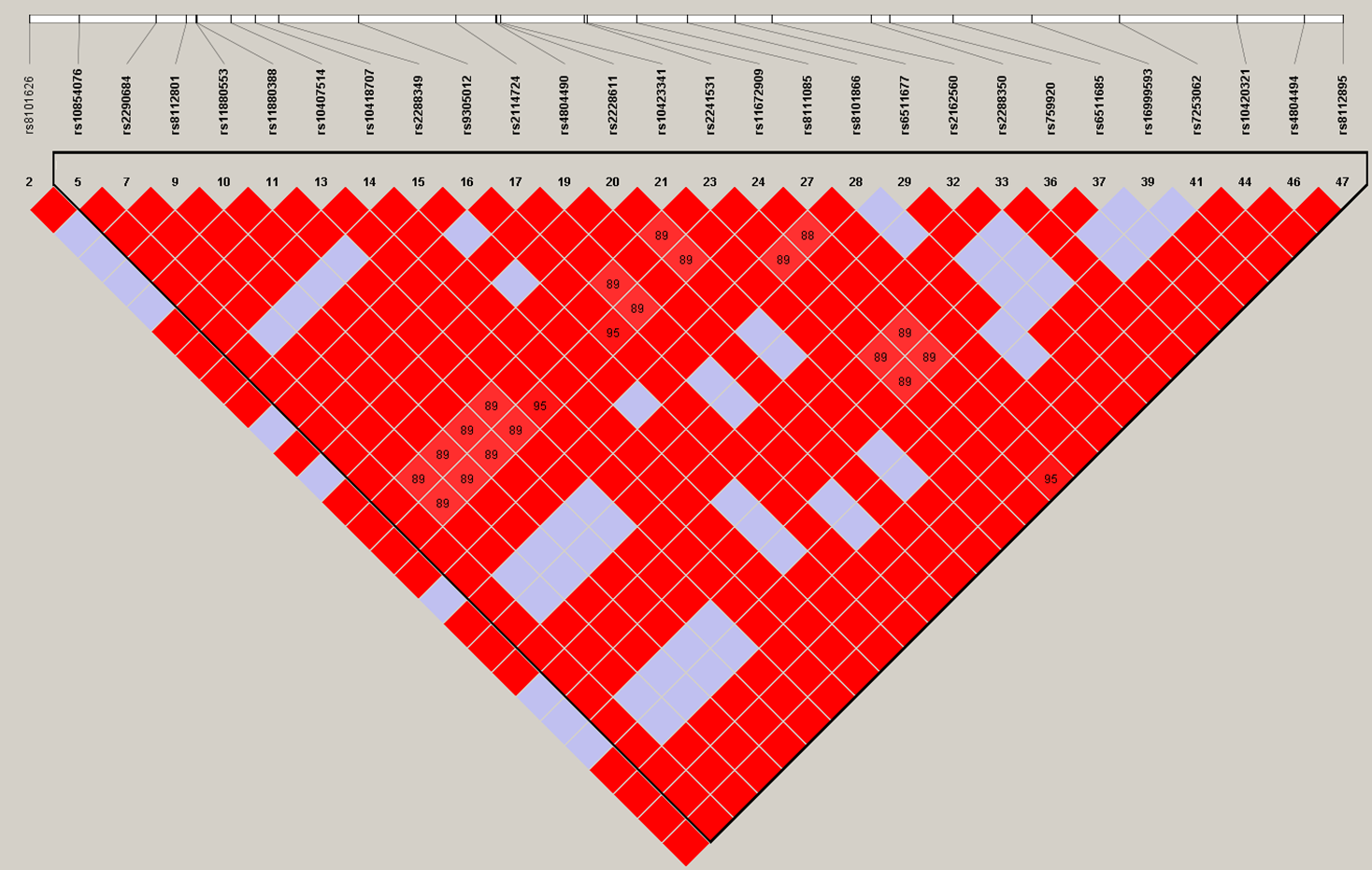


**Figure S1. Linkage disequilibrium plot of SNPs in *DNMT1*.** Dark red represents complete linkage; number in red box indicates r^2^ × 100; light blue represents incomplete linkage; number below each SNP indicates position. DNMT1, DNA methyltransferase 1; SNP, single nucleotide polymorphism.


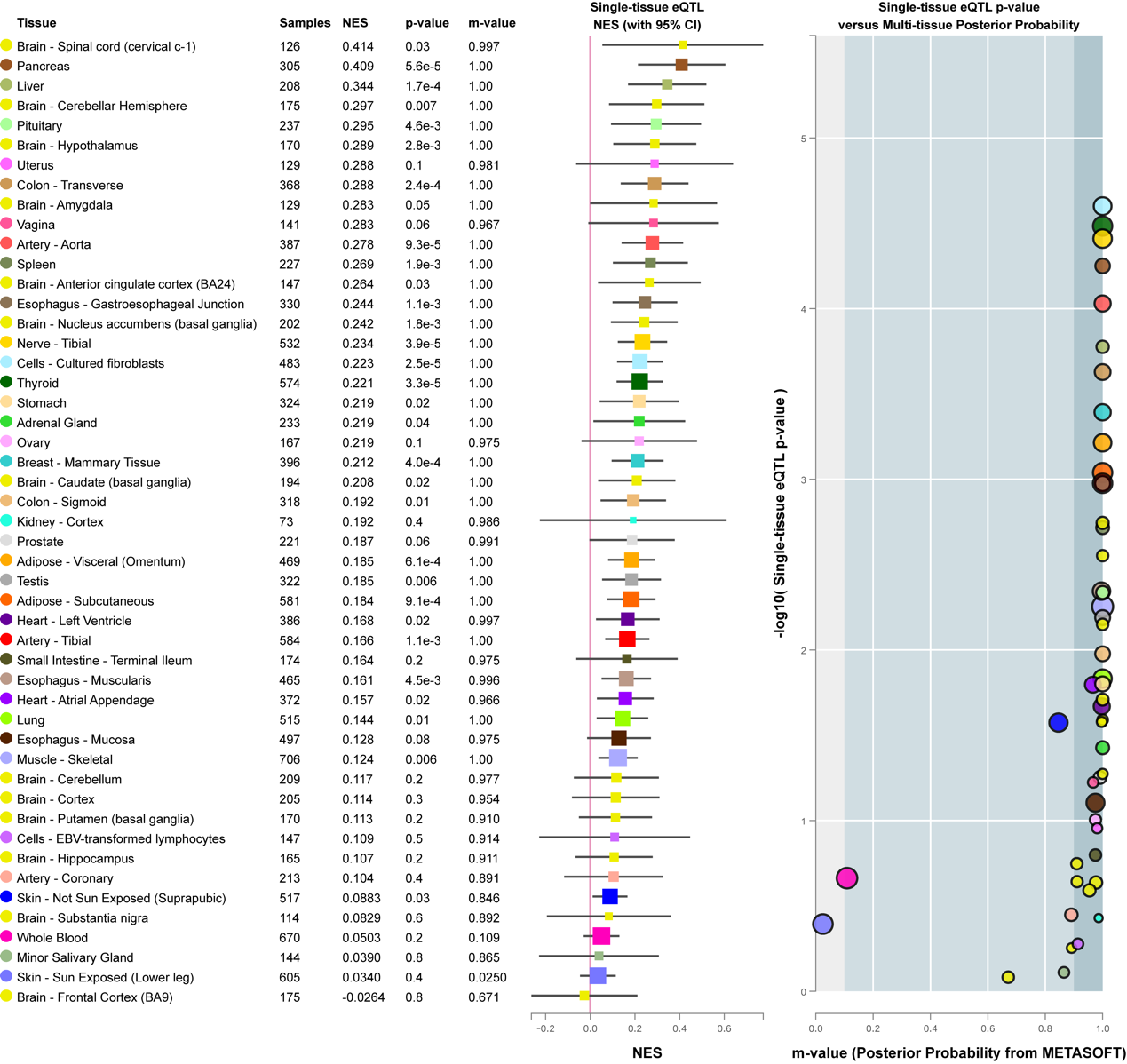


**Figure S2.** Multi-tissue eQTL correlation analysis between rs9305012 and the *P2RY11* expression. The plot was generated from GTEx. Significance was considered at *P* < 0.05. CI, confidence interval; eQTL, expression quantitative trait locus; NES, normalized effect size; different color represents different tissue; m-value, the posterior probability that an eQTL effect exists in each tissue tested in the cross-tissue meta-analysis.


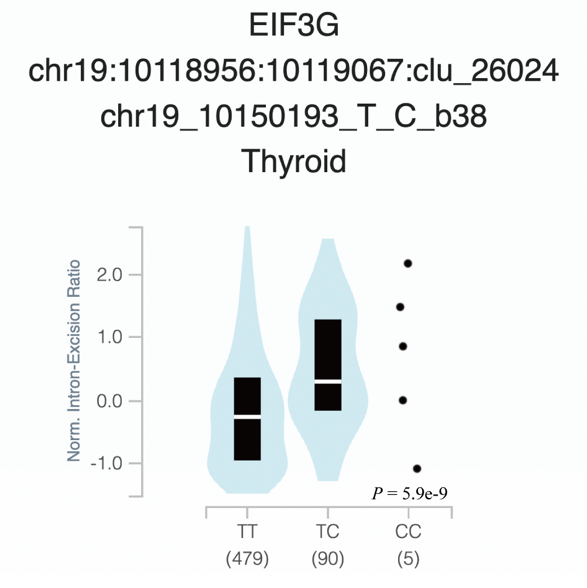


**Figure S3.** sQTL violin plots for rs9305012 on intron-excision ratio of *EIF3G* in human thyroid tissue. Data were obtained from GTEx. Significance was considered at *P* < 0.05. EIF3G, eukaryotic translation initiation factor 3 subunit G; sQTL, splicing quantitative trait locus.
